# Correlation between PET/CT and CT in the staging prior to the treatment of head and neck squamous cell carcinoma

**DOI:** 10.1016/j.bjorl.2019.06.004

**Published:** 2019-07-06

**Authors:** Fernando García-Curdi, Yolanda Lois-Ortega, Ana Muniesa-del Campo, Amaranta McGee-Laso, José Miguel Sebastián-Cortés, Héctor Vallés-Varela, Julio José Lambea-Sorrosal

**Affiliations:** a Lozano Blesa University Hospital, Department of Otorhinolaryngology, Zaragoza, Spain; b Ernest LLuch Martín Hospital, Department of Otorhinolaryngology, Calatayud, Spain; c University of Zaragoza, Faculty of Veterinary Sciences, Department of Animal Pathology, Zaragoza, Spain; d University Care Complex of León, Department of Preventive Medicine, León, Spain; e Lozano Blesa University Hospital, Department of Medical Oncology, Zaragoza, Spain

**Keywords:** Head and neck squamous cell carcinoma, Staging, Correlation, Positron emission computed tomography, Computed tomography

## Abstract

**Introduction:**

Head and neck squamous cell carcinoma is the seventh most common malignant tumor. The advances in treatment have improved the global survival rates in the past years, although the prognosis is still grave.

**Objective:**

The aim of the present study is to evaluate the correlation between positron emission computed tomography and computed tomography at the time of staging a previously untreated head and neck squamous cell carcinoma, and to determine which of the two imaging techniques gives us more information at the time of initial diagnosis.

**Methods:**

Data from all patients diagnosed in our hospital of head and neck squamous cell carcinoma by a biopsy of any location or unknown primary tumor was collected, between January 2012 and July 2017. In all cases, computed tomography and positron emission computed tomography were performed with a maximum of 30 days difference between them and patients had not received any prior treatment to staging. The stage given to each case was compared based solely on the physical examination, only on the computed tomography/positron emission computed tomography, with respect to the stage given by the tumor board, observing the concordance obtained through Cramer's V statistical test.

**Results:**

We performed a comparative analysis obtaining a correlation of 0.729 between the stage given by the tumor board and the one assigned based on the physical examination without imaging techniques. When only using computed tomography as an imaging method, the correlation was 0.848, whereas with only the use of positron emission computed tomography it was estimated at 0.957. When comparing the statistical association between staging using exclusively one of the two imaging techniques, correlation was 0.855.

**Conclusion:**

Positron emission computed tomography is useful for the diagnosis of head and neck squamous cell carcinoma, improving the patient's staging especially when detecting cervical and distant metastases. Therefore, we consider that the use of positron emission computed tomography for the staging of patients with head and neck squamous cell carcinoma is a diagnostic test to be considered.

## Introduction

Head and neck squamous cell carcinoma (HNSCC) is the seventh most common malignant tumor, its incidence growing worldwide, with approximately 600,000 new cases diagnosed per year,^1^ representing up to 4% of new cancer diagnoses, and the eighth cause of death by cancer.^2^

The advances in treatment have improved the global survival rates in recent years, although the prognosis is still grave. Global survival at 5 years varies between 45–55%, although patients diagnosed in early stages have up to 80% survival rates depending on the location of the tumor; 60% of cases are diagnosed at advanced stages, due to the lack of anatomic barriers, the abundance of lymphatic drainage in the area and the infiltrative growth pattern.^3^

Initial staging of HNSCC includes: physical examination (inspection, palpation and rhinolaryngoscopy), computed tomography (CT) and/or magnetic resonance imaging (MRI), in order to evaluate the local extension of the primary tumor and the involvement of regional lymphatic nodes.

Several studies have reported the benefit of positron emission tomography/computed tomography (PET/CT) using the 18F-fluorodeoxyglucose analog (18F-FDG PET/TC), in comparison with conventional imaging techniques used in the detection of cervical lymph nodes invasion and distant metastasis^4^ since it is a complete body imaging technique.^5^, ^6^, ^7^ Nowadays, the use of PET/CT is recommended in cases of doubtful regional lymph node involvement, and in unknown primary tumor (UPT),^8^ since this technique can detect up to 1/3 of primary tumors, changing the clinical management of the disease.^9^

PET/CT is used more every day in the diagnosis, classification, staging and evaluation of the response to treatment in a variety of malignant tumors. It has been suggested that PET/CT could be more precise than CT and/or MRI in identifying cervical lymph node metastases in HNSCC because it is considered to have a higher sensitivity,^10^ although some studies suggest that the PET technique does not detect regional and distant metastases of small volume.^11^

Despite the improvement in the detection of regional pathologic adenopathies, the detection of distant metastases and the discovery of second primary tumors, there is limited data about the impact of PET/CT in the variation of staging in comparison with conventional imaging techniques, since the studies in the medical literature are not conclusive due to small sample sizes and the use of a variety of methods to determine the performance of PET/CT.^12^

The objective of this study is to evaluate the correlation between CT and PET/CT in the staging of HNSCC given by the Multidisciplinary Tumor Board of the Hospital Clínico Universitario Lozano Blesa of Zaragoza, in order to clarify which of the techniques results in more precise information.

## Methods

Data from 44 patients was collected between January 2012 and July 2017, after the approval of the Investigation Ethics Committee of the Region of Aragón (CEICA) and by the management board of the Hospital Clínico Universitario Lozano Blesa.

The inclusion criteria were: diagnosis by biopsy of HNSCC in our hospital during the period of study in any location (paranasal sinuses, nasopharynx, oral cavity, oropharynx, hypopharynx, larynx) or UPT. Patients were required to have a CT and a PET/CT performed in a maximum period of 30 days between techniques, and no treatment before staging. Stages were assigned according to the American Joint Committee on Cancer Staging Manual (AJCC) (7th edition) classification. All patients who did not meet these criteria were excluded from the study.

It is an observational study with historical data, performed in a tertiary hospital. All patients were subject to physical examination (inspection, palpation and rhinolaryngoscopy), cervicothoracic CT with contrast and a PET/CT. The imaging protocol (CT and PET/CT) was as follows:

CT scan (neck and chest) was performed after an intravenous iodine contrast injection. The first one was centered in the head and neck region (from the skull base to the thoracic inlet with a slice thickness of 1 mm). The second one was performed from the thoracic inlet to the adrenal glands (3 mm slice thickness) just after repositioning the arms (from below to above the shoulders).

PET-CT technique: after patients had fasted for at least 6 h and 60 min before the scan, they were given an intravenous injection of fluorodeoxyglucose (FDG) using a dosage of 3–4 MBq/kg. Images were acquired from the head to the mid-thighs in axial plane, and then reconstructed in coronal and sagittal planes.

A biopsy of the lesion was performed in order to confirm the HNSCC diagnosis. The data was then presented to the Multidisciplinary Tumor Board of the Hospital Clínico Universitario Lozano Blesa. The board is formed by otorhinolaryngologists, medical oncologists, radiotherapy oncologists, anatomopathologists, nuclear medicine specialists and radiologists. After the presentation of every case, including the physical examination (performed by an otorhinolaryngologist) and the diagnostic tests (evaluated by radiologists and specialists in nuclear medicine, depending on the test performed, radiologists for CT and nuclear medicine specialists for PET/CT), a TNM stage was established following the AJCC classification.

The stage given to every case was compared with the stage given by the Tumor Board using only the physical exam, only the CT and only the PET/CT, observing the concordance obtained through Cramer’s V statistic test. In addition, we performed an individualized correlation analysis between the diagnostic methods of T (primary tumor), N (pathological adenopathies) and M (distant metastases), grouping tumors of all locations.

Data was analyzed using IBM SPSS 19.0 for Windows (IBM Corp., Armonk, NY, EE UU). The association between the different techniques was established using the likelihood ratio statistic test, and quantified using Cramer’s V test. Alpha was established at 0.05, corresponding to a 95% Confidence Interval.

## Results

The total number of patients analyzed from January 2012 to July 2017 was 44, with a mean age of 65.74 years, being mainly males. The characteristics are summarized in Table 1.

**Table 1:** Patients characteristics.

Characteristics	Number of patients (%)
Sex	
Men	40 (90.9%)
Women	4 (9.1%)
Age	
Median age	65.74 (min. 30‒max. 87)
Location of the primary tumour	
Paranasal sinuses	1 (2.3%)
Nasopharynx	6 (13.6%)
Oropharynx	8 (18.2%)
Hipopharynx	3 (6.8%)
Larynx	22 (50%)
Supraglottis	19 (43.2%)
Glottis	3 (6.8%)
UPT	3 (6.8%)
Total	
Stage	
I	1 (2.27%)
II	6 (13.64%)
III	10 (22.73%)
IV	24 (54.54%)
UPT	3 (6.82%)

During the comparative analysis of the staging obtained through physical examination and the one given by the Tumor Board we determined a statistical significance (*p* < 0.001) and a correlation coefficient of V = 0.729. In approximately 50% of Stages I, II and III cases, there is an underestimation of staging in the medical consult, compared with the staging assigned by the Tumor Board, while in Stage IV cases the clinical diagnosis was correct 100% of times. Without any imaging techniques, 31.81% of HNSCC in our series are understaged, compared with the Tumor Board diagnosis, while 2.12% are overstaged (Table 2).

**Table 2:** Correlation between the stagings obtained through physical examination and the one given by the Tumor Board.

	Tumor board stage						
		UPT	I	II	III	IV	Total
Physical examination stage	UPT	3	0	0	0	0	3
	I	0	1	1	0	0	2
	II	0	0	4	1	2	7
	III	0	0	1	9	10	20
	IV	0	0	0	0	12	12
	Total	3	1	6	10	24	44
	Cramer’s V 0.729, *p* < 0.001.						

	Tumor board T									
		UPT	T1	T1a	T1b	T2	T3	T4a	T4b	Total
Physical examination T	UPT	3	0	0	0	0	0	0	0	3
	T1	0	1	0	0	1	0	0	0	2
	T1a	0	0	1	0	0	0	0	0	1
	T1b	0	0	0	0	0	0	0	0	0
	T2	0	0	0	0	11	2	1	0	14
	T3	0	0	0	0	1	19	4	0	24
	T4a	0	0	0	0	0	0	0	0	0
	T4b	0	0	0	0	0	0	0	0	0
	Total	3	1	1	0	13	21	5	0	44
	Cramer’s V 0.878, *p* < 0.001.									

	Tumor board N								
		N0	N1	N2	N2a	N2b	N2c	N3	Total
Physical examination N	N0	10	1	0	1	2	3	0	17
	N1	0	5	0	0	0	4	0	9
	N2	0	0	2	0	0	0	0	2
	N2a	0	0	0	0	2	2	0	4
	N2b	0	0	0	0	5	3	0	8
	N2c	0	0	0	0	0	3	0	3
	N3	0	0	0	0	0	0	1	1
	Total	10	6	2	1	9	15	1	44
	Cramer’s V 0.719, *p* < 0.001.								

When analyzing TNM separately, we can observe that physical examination as the only diagnostic method tends to understage the primary tumor, especially in advanced cases (T3 and T4a), with a correlation of 0.878. When analyzing cervical adenopathies the same pattern is observed, and in no case physical examination overdiagnosed pathological adenopathies, while in 18 of the 44 total cases, N was underestimated, especially from N2b and over (Cramer’s V 0.719). In the case of distant metastases, they are undetectable unless imaging techniques are performed (Table 2).

When we examine the correlation between the stage obtained by physical examination and CT, and the one given by the Tumor Board, we obtain a statistical significance (*p* < 0.001), and a correlation coefficient of V = 0.848. In 5 of the 44 cases (11.36% of our series), CT did not clearly show the primary tumor, 3 of them being UPT. In our sample, when excluding PET/CT of the diagnostic tests, the stage is underestimated 18.18% of times, and overestimated 4.54% of occasions (Table 3).

**Table 3:** Correlation between the staging obtained by physical examination and CT, and the one given by the Tumor Board.

	Tumor board stage							
		Tumor is not detected	UPT	I	II	III	IV	Total
Physical examination + CT stage	Tumor is not detected	0	0	0	0	0	2	2
	UPT	0	0	0	0	0	0	3
	I	0	0	1	0	0	0	1
	II	0	0	0	5	2	1	8
	III	0	0	0	0	7	3	10
	IV	0	0	0	1	1	18	20
	Total	0	3	1	6	10	24	44
	Cramer’s V 0.848, *p* < 0.001.							

	Tumor board T										
		Tumor is not detected	UPT	T1	T1a	T1b	T2	T3	T4a	T4b	Total
Physical examination + CT T	Tumor is not detected	0	0	0	0	0	1	1	0	0	2
	UPT	0	3	0	0	0	0	0	0	0	3
	T1	0	0	1	0	0	0	0	0	0	1
	T1a	0	0	0	1	0	0	0	0	0	1
	T1b	0	0	0	0	0	0	0	0	0	0
	T2	0	0	0	0	0	11	1	0	0	12
	T3	0	0	0	0	0	0	19	0	0	19
	T4a	0	0	0	0	0	1	0	5	0	6
	T4b	0	0	0	0	0	0	0	0	0	0
	Total	0	3	1	1	0	13	21	5	0	44
	Cramer’s V 0.912, *p* < 0.001.										

	Tumor board N								
		N0	N1	N2	N2a	N2b	N2c	N3	Total
Physical examination + CT N	N0	10	3	0	1	1	0	0	15
	N1	0	3	0	0	0	2	0	5
	N2	0	0	2	0	0	0	0	2
	N2a	0	0	0	0	1	0	0	1
	N2b	0	0	0	0	6	5	0	11
	N2c	0	0	0	0	1	8	0	9
	N3	0	0	0	0	0	0	1	1
	Total	10	6	2	1	9	15	1	44
	Cramer’s V 0.742, *p* < 0.001.								

The separated analysis of TNM indicates a good correlation for the diagnosis of the primary tumor using only the CT as imaging (Cramer’s V = 0.912). In 38 of 44 cases there was a concordance, 3 cases being UPT and not visualizing the primary tumor in 2 cases. Regarding N analysis, we observe that the CT tends to understage these lesions (Cramer’s V = 0.742), especially in advanced stages (N2b and over, the most errors occurring when N2c stage). As in the case before, we cannot assess distant metastases, since the CT performed is not an entire body test (Table 3).

When studying the correlation obtained between the staging based on physical examination and PET/CT and the one given by the Tumor Board, we obtain a statistical significance (*p* < 0.001), and a correlation coefficient of V = 0.957. When excluding CT from our diagnostic tests, and using PET/CT as the only imaging technique, we obtain a 4.54% overestimation in the stage (2 patients of the series), while no underestimation was obtained compared with the Tumor Board (Table 4).

**Table 4:** Correlation between the staging obtained by physical examination and PET/CT, and the one given by the Tumor Board.

	Tumor board stage						
		UPT	I	II	III	IV	Total
Physical examination + PET/CT stage	UPT	3	0	0	0	0	3
	I	0	1	0	0	0	2
	II	0	0	5	0	0	5
	III	0	0	1	9	0	9
	IV	0	0	1	1	24	26
	Total	3	1	6	10	24	44
	Cramer’s V 0.957, *p* < 0.001.						

	Tumor board T										
		Tumor is not detected	UPT	T1	T1a	T1b	T2	T3	T4a	T4b	Total
Physical examination + PET/CT T	Tumor is not detected	0	0	0	0	0	1	1	0	0	0
	UPT	0	3	0	0	0	0	0	0	0	3
	T1	0	0	1	0	0	0	0	0	0	1
	T1a	0	0	0	1	0	0	0	0	0	1
	T1b	0	0	0	0	0	0	0	0	0	0
	T2	0	0	0	0	0	12	0	0	0	12
	T3	0	0	0	0	0	0	20	0	0	20
	T4a	0	0	0	0	0	1	1	5	0	7
	T4b	0	0	0	0	0	0	0	0	0	0
	Total	0	3	1	1	0	13	21	5	0	44
	Cramer’s V 0.960, *p* < 0.001.										

	Tumor board N								
		N0	N1	N2	N2a	N2b	N2c	N3	Total
Physical examination + CT N	N0	9	0	0	1	0	0	0	10
	N1	0	6	0	0	0	0	0	6
	N2	0	0	2	0	0	0	0	2
	N2a	0	0	0	0	1	0	0	1
	N2b	1	0	0	0	8	0	0	9
	N2c	0	0	0	0	0	15	0	15
	N3	0	0	0	0	0	0	1	1
	Total	10	6	2	1	9	15	1	44
	Cramer’s V 0.897, *p* < 0.001.								

	Tumor board M			
		M0	M1	Total
Physical examination + CT M	M0	38	0	38
	M1	1	5	6
	Total	39	5	44
	Cramer’s V 0.901, *p* < 0.001.			

In this case, when analyzing separately the TNM, we observe a good correlation of the T (Cramer’s V = 0.960). Physical examination and PET-CT never underestimated the primary tumor, and in 2 cases it was overstaged. Regarding the N, there is a correlation of 0.897, with little discrepancies in over and understanging of cervical pathological adenopathies. When assessing distant metastases, only in once case there were differences, when PET-CT showed a false positive (Cramer’s V = 0.901) (Table 4).

Ultimately, if we compare the stages of our sample based on the physical examination and PET/CT regarding the physical examination and CT, we obtain a statistical significance (*p* < 0.001), and a correlation coefficient of V = 0.855. CT did not show the primary tumor in 5 patients, 3 being UPT. When using CT, 1 case was given a higher stage compared to the use of PET/CT; representing 2.27% of our sample and 9 cases (20.45%) were given a lower stage (Table 5).

**Table 5:** Correlation between the staging obtained by the physical examination and PET/CT with respect to the stage obtained through physical examination and CT.

	Physical examination + CT stage							
		Tumor is not detected	UPT	I	II	III	IV	Total
Physical examination + PEC/CT stage	Tumor is not detected	0	0	0	0	0	0	0
	UPT	0	3	0	0	0	0	3
	I	0	0	1	0	0	0	1
	II	0	0	0	5	0	0	5
	III	0	0	0	2	6	1	9
	IV	2	0	0	1	4	19	26
	Total	2	3	1	8	10	20	44
	Cramer’s V 0.855, *p* < 0.001.							

	Physical examination +/CT T										
		Tumor is not detected	UPT	T1	T1a	T1b	T2	T3	T4a	T4b	Total
Physical examination + PET/CT T	Tumor is not detected	0	0	0	0	0	0	0	0	0	0
	UPT	0	3	0	0	0	0	0	0	0	3
	T1	0	0	1	0	0	0	0	0	0	1
	T1a	0	0	0	1	0	0	0	0	0	1
	T1b	0	0	0	0	0	0	0	0	0	0
	T2	1	0	0	0	0	11	0	0	0	12
	T3	1	0	0	0	0	1	18	0	0	20
	T4a	0	0	0	0	0	0	1	6	0	7
	T4b	0	0	0	0	0	0	0	0	0	0
	Total	2	3	1	1	0	12	19	6	0	44
	Cramer’s V 0.915, *p* < 0.001.										

	Physical examination + CT N								
		N0	N1	N2	N2a	N2b	N2c	N3	Total
Physical examination + PET/CT N	N0	10	0	0	0	0	0	0	10
	N1	3	3	0	0	0	0	0	6
	N2	0	0	2	0	0	0	0	2
	N2a	0	0	0	0	1	0	0	1
	N2b	2	0	0	0	6	1	0	9
	N2c	0	2	0	0	5	8	0	15
	N3	0	0	0	0	0	0	1	1
	Total	15	5	2	1	11	9	1	44
	Cramer’s V 0.832, *p* < 0.001.								

Regarding the comparison between diagnostic methods, in the case of the primary tumor analysis there is a correlation of 0.915. There were 4 occasions when there was an underdiagnosis using CT, not localizing the primary tumor in 2 of them. When analyzing the N we observe discrepancies between CT and PET/CT, because of primarily CT’s underdiagnosis. PET-CT can diagnose metastatic adenopathies that CT does not detect, and the main difference is observed in the N2c stage (when detecting contralateral adenopathies). The correlation of both diagnostic methods is 0.832. As mentioned above, CT does not trace the whole body, so we did not compare distant metastases (Table 5).

## Discussion

In the present study we compare the utility of PET/CT opposing CT in the staging of HNSCC. Different studies showing the utility of PET/CT in the diagnosis of cervical ipsilateral and contralateral adenopathies have been performed, showing a higher sensitivity than CT or MRI.^13^

Currently, imaging techniques are essential for the diagnosis, since they are fundamental for the staging of the tumor, especially in the early phases of the disease, when the physical exam is insufficient due to the difficulty in detecting pathologic adenopathies only through palpation, or assessing tumor infiltration using a fibroscope.

Actual clinical guides recommend PET/CT as an option in the advanced stages of the disease, due to low performance of PET/CT in patients with Stage I or II. This is the reason why in our sample there are not many patients in early stages of the disease, but according to the analyzed data, we can observe that when using PET/CT in the diagnosis of HNSCC we obtain a statistically significant higher precision than when using CT, both in the initial and late stages of the disease.

Likewise, the use of PET/CT is indisputable in UPT when localizing the primary tumor. In our series, PET/CT identified the primary tumor in 3 out of 9 cases where the CT was not successful. In other series published in the literature, PET/CT is capable of finding the primary tumor in approximately 30% of cases,^14^, ^15^ and though in our study the percentage is higher, it may be due to our sample size.

In addition, the use of PET/CT, being a whole imaging body technique, identified distant metastasis in 6 cases, while cervicothoracic CT only showed one case (pulmonary metastasis). In general, the use of PET/CT reduces the number of unnecessary surgical interventions and optimizes the surgical indications in these patients.^16^, ^17^

Other published studies have demonstrated a higher precision in staging head and neck tumors when using PET/CT,^5^, ^18^ as well as its utility in diagnosing retrophanryngeal metastatic adenopathies, having a great impact in the prognosis and therapeutic management of these patients.^19^ However, the reported results of patients who have benefited from a change in stage by CT exam vary: Keyes et al. published a retrospective cohort study evaluating the frequency with which PET contributed to new significant information in comparison with standardized evaluation, and reported that only 2% of patients benefited from the use of the PET exam.^20^ On the contrary, Fleming et al. retrospectively studied the role of PET/CT in 123 patients without previously treated malignant head and neck tumors, and found an inadequate treatment in 30.9% of patients (38 out of 123).^21^

The limitations of our study are the retrospective design and the sample size. However, the results are statistically significant. We report the utility of incorporating PET/CT to the diagnosis of patients suffering HNSCC, improving the staging and the therapeutic plan.

We have not analyzed the cost-effectiveness of using PET/CT routinely, but there are studies in the literature affirming that the savings in unnecessary surgical interventions are higher than the costs of PET/CT.^22^ Others have demonstrated that the use of PET/CT previous to treatment is the most effective diagnostic method without generating any extra costs.^23^

According to the results of our study, we can determine that the use of PET/CT improves the staging of patients with head and neck tumors, aiding in a better therapeutic plan for each patient. We consider that these results are representative of the usual clinical practice.

## Conclusions

PET/CT is useful in the diagnosis of HNSCC, being more precise than CT, improving the patient staging especially when detecting cervical and distant metastases. Therefore, we consider that the use of PET/CT in the staging of patients with HNSCC should be considered. The therapeutic impact that the change in staging has entailed in these patients has not yet been evaluated, which can be a future line of investigation for our working group.

## Conflicts of interest

The authors declare no conflicts of interest.
